# Effects of LaCoO_3_ perovskite nanoparticle on *Daphnia magna*: accumulation, distribution and biomarker responses

**DOI:** 10.1039/c9ra03513c

**Published:** 2019-08-08

**Authors:** Tingting Zhou, Lili Zhang, Ying Wang, Qian Mu, Jingyu Yin

**Affiliations:** School of Space and Environment, Beihang University No. 37, XueYuan Road, Haidian District Beijing 100191 PR China yingw@buaa.edu.cn; Shanghai Institute of Applied Physics, Chinese Academy of Sciences No. 239, Zhangheng Rd, Pudong District Shanghai P. R. China

## Abstract

Perovskite nanomaterials (PNMs) have been shown to be promising materials for the effective replacement of conventional energy source materials. With the increasing use of PNMs, they will inevitably enter aquatic environments, giving rise to concerns regarding the environmental impact of PNMs. To fill up the gap in information about the environmental effect of PNMs, *Daphnia magna* was exposed to a typical PNM LaCoO_3_ for 48 h, to assess temporal patterns in PNM bioaccumulation and distribution. Synchrotron radiation based micro X-ray fluorescence spectroscopy (μ-XRF) was used to investigate the time dependent spatial distribution of LaCoO_3_. Reactive oxygen species (ROS), superoxide dismutase (SOD) and Na^+^/K^+^-adenosine triphosphatase (ATPase) were measured as key biomarkers. The results showed that oxidative stress was observed at both LaCoO_3_ concentrations and Na^+^/K^+^-ATPase was inhibited by high levels of LaCoO_3_. The mode of action of LaCoO_3_ was mainly dependent on the metal forms. At low LaCoO_3_ levels, food ingestion was the main entry pathway into organisms and LaCoO_3_ nanoparticle aggregates accumulated in the gut area. At high LaCoO_3_ levels, both waterborne and dietary uptake was observed and the gut and thoracic limbs were the main target sites for LaCoO_3_ nanoparticle aggregates and dissolved ions, respectively. LaCoO_3_ was not found to translocate in daphnids during the 48 h exposure period at either concentration, suggesting that internalization did not occur. These findings help further our understanding of the fate of PNMs in aquatic organisms, as well as the associated biological responses to PNM exposure.

## Abstract 2

The instability of PNMs in water is of environmental concern. This study shows that in daphnids over 48 h, the mode of action of a representative PNM LaCoO_3_ is dependent on Co species, which results in the differences in uptake, accumulation, distribution and toxicity.

## Graphical abstract

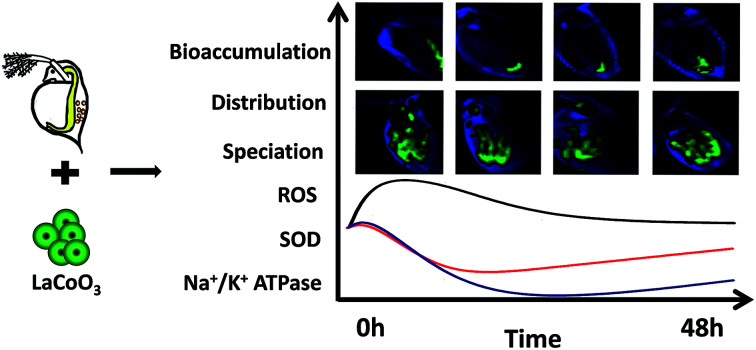

## 1. Introduction

The global energy crisis is one of the most important problems faced by society to date, with fossil fuel resources being both limited and highly damaging to the environment. Recent research achievements have established novel energy source materials using perovskite nanomaterials (PNMs).^1–3^ As the use of PNMs increases, they will inevitably enter aquatic environments and be taken up by aquatic organisms, which can then exert adverse effects on aquatic biota. Therefore, there is increasing concern about the environmental impact of PNMs when used in large-scale applications.^4,5^ In the past decade, property–activity relationships were established to aid understanding of the toxicity mechanism of nanoparticles. For PNMs, which have both the structure of perovskite and properties of nanoparticles (NP), it is still largely unknown what hazard they will present to ecological organisms.

Although the study about the environmental effect of PNMs was limited, the effect and mechanism about NPs to aquatic organisms are plentiful. Triggering reactive oxygen species (ROS) mediated oxidative stress is the most widely accepted toxic mechanism for NPs and results in proteomic and non-proteomic responses.^6–8^ These responses could be antioxidant enzymes such as superoxide dismutase (SOD) and catalase (CAT), which play important roles in maintaining a balance between prooxidant and antioxidant processes. When the level of ROS production overwhelms the defensive capacity of organisms, significant oxidative damage occurs, including enzyme inactivation, DNA damage and lipid peroxidation.^9^ Furthermore, NPs or their dissolved parts can further impair membrane functions, inactivate membrane-bound enzymes, and increase membrane permeability.^10^ Na^+^/K^+^-ATPase is an inner membrane protein that exists on the plasma membrane to hydrolyze ATP to obtain energy and plays a major role in cellular regulation of ionic homeostasis.^11^ It exists extensively in aquatic animals and is susceptible to membrane damage and evidence points to a role of free radicals in its activity disruption.^12^ Any changes in organism biochemistry could have a negative effect on the well-being of the organism and, if prolonged, could ultimately lead to death.

Once released into aquatic environments, nanoparticles are subject to processes (*e.g.*, dissolve to ions; aggregate/agglomerate; bind with other ions, molecules or particular organic matter; or remain in nano-form), which influence their behavior, reactivity, uptake by organisms and toxicity. Among these issues, uptake and bioaccumulation of NPs have been proposed to be a prerequisite for various adverse effects.^13,14^ According to recent literatures, the uptake of NPs is strongly affected by their physicochemical properties.^15,16^ In the case of *D. magna*, size, speciation, concentration, surface charge and crystalline phase were manifested to affect the uptake of NPs. These observations can be related to the way of filter feeding of *D. magna*, which collect particles or ions through waterborne or dietary, *via* their thoracic limbs and filter apparatus. NPs mainly accumulated in the gut and was difficult to be eliminated completely by *D. magna*^17–20^ and thus produced long-term toxic effect. A small part of NPs were also observed accumulated in the brood chamber and abdominal area^19,21^ and may directly damage the organs or certain physiological functions of daphnids. Despite the numerous studies of NPs toxicokinetics that have been conducted, including bioavailability, accumulation, and depuration of NPs in daphnids after it exposure, there is a critical gap in information about temporal patterns of distribution and chemical speciation of NPs, which is essential to understand biochemical processes in organisms and shown to affect the toxic effect. However, due to the lack of non-destructive characterization methods, time dependent spatial distribution of NPs in living organisms was rarely studied *in situ*. The development of synchrotron radiation technologies such as microbeam synchrotron radiation X-ray fluorescence spectroscopy (μ-SRXRF), have enabled advanced spatial distribution analysis at a high spatial resolution (μm or nm scale). Synchrotron radiation technologies have been effectively used to study the uptake and transport of NPs in cell and plant systems.^22,23^ However, at present there is little data available on temporal patterns of NP distribution and biotransformation *in vivo*.

In this study, *D. magna* were exposed to two concentrations of LaCoO_3_ and its corresponding cation (Co^2+^ ions) for different periods of time (0, 3, 12, 24 and 48 h). The accumulation and internal distribution patterns of La and Co were investigated in LaCoO_3_-exposed daphnids using μ-SRXRF spectroscopy. Changes in biomarkers were also investigated in *D. magna*, in relation to temporal and spatial changes in the distribution of LaCoO_3_, including ROS and the antioxidant biomarkers SOD and Na^+^/K^+^-ATPase. The overall objective of this study was to determine the time-dependent toxicological modes of action of LaCoO_3_.

## 2. Materials and methods

### 2.1 Preparation and characterization of LaCoO_3_ nanoparticle

LaCoO_3_ nanoparticles were prepared according the method reported by Chandradass *et al.*, (2014) with some modifications:^24^ certain amount of La(NO_3_)_3_·6H_2_O and Co(NO_3_)_2_·6H_2_O were weighed precisely according to the atomic ratio of the target product and dissolved in 80 mL ethanol, then ammonia water were added dropwise under stirring to form La(OH)_3_ and Co(OH)_2_ and the final pH was 9. The suspension were stirred for two more hours. The precipitate were washed with ethanol for several times and dried at 80 °C for 48 h and finally calcined at muffle at 600 °C for 2 h. The crystal structure were characterized using X-ray diffraction (XRD), which was done recorded using a Bruker D8 Advance diffractometer equipped with Cu-Kα source. The data were collected by using step scanning at 2*θ* = 10–90° with step size of 0.058. The particle sizes were characterized using transmission electron microscopy (TEM; Hitachi 7500).

### 2.2 Characterization of LaCoO_3_ nanoparticle exposure medium

The simplified Elendt M7 medium (SM7), which consists of CaCl_2_ (293.8 mg L^−1^), MgSO_4_ (123.3 mg L^−1^), K_2_HPO_4_ (0.184 mg L^−1^), NaNO_3_ (0.274 mg L^−1^), NaHCO_3_ (64.8 mg L^−1^), Na_2_SiO_3_ (10 mg L^−1^), H_3_BO_3_ (0.175 mg L^−1^), KCl (5.8 mg L^−1^), and KH_2_PO_4_ (0.143 mg L^−1^), and pH of 7.8–8.2 recommended by standard testing guidelines (OECD, 2012) was select as the test medium and used in all experiments. LaCoO_3_ were added to a SM7 medium to obtain a stock suspension of 100 ppm, which was stirred for 2 hours and then sonicated for 30 min at 100 W (KQ-500DB, Kun Shan, China). Samples were then diluted in SM7 medium to obtain nominal concentrations of 5 and 50 ppm LaCoO_3_.

For point of zero charge measurements, the zeta potential of LaCoO_3_ as a function of pH, was measured in ultrapure water using a Zetasizer analyzer (Nano ZEN 3600, Malvern, UK) at 25 °C. Analysis was conducted on 50 ppm LaCoO_3_, with 0.1 M nitric acid and 0.1 M sodium hydroxide used to achieve desired pH levels. All measurements were made 30 minutes after pH conditions were altered.

To obtain the actual concentration of LaCoO_3_ in suspension over time, 1 mL samples were taken from exposure suspensions at the same depth at 0, 3, 6, 12, 24 and 48 h and then acidified with 2 mL HNO_3_. La and Co concentrations were analyzed by inductively coupled plasma mass spectrometry (ICP-MS, VGPQ2 TURBO, USA).

The dissolution of LaCoO_3_ was investigated prior to the biotoxicity test. 1 mL samples of the exposure suspension supernatant were ultrafiltered using centrifuge tubes with a nominal molecular weight limit of 10 kDa (Millipore, USA). Samples were centrifuged at 4000 *× g* for 60 min at 4 °C. A subsample from the filtrate was acidified with 2% HNO_3_ and stored in the dark prior to analysis, with concentrations of La and Co in the supernatant determined by ICP-MS.

### 2.3 *D. magna* toxicity assays

#### 2.3.1 *D. magna* culture and toxicity assays

*D. magna* were maintained for 2 years and were cultured at 23.5 °C under a 16 : 8 light–dark cycle in natural water, collected from Hue Qi Ying Bridge (China, 39°58′5.58′′N, 116°16′53.3′′E). The water was renewed three times a week, and the *D. magna* density was 1 individual per 10 mL. *Scenedesmus obliquus* were fed daily to *D. magna* at a concentration of 1–2 × 10^5^ cells per mL. Monthly check for sensitivity of *D. magna* to potassium dichromate, making sure the 50% effective concentration (EC50) is within the limits set by the Organisation for Economic Co-operation and Development (OECD) Guideline 202.

5 ppm and 50 ppm LaCoO_3_ samples were prepared by dispersing samples in SM7 using ultrasonic treatment at an intensity of 100 W for 20 min in an attempt to obtain the optimal particle dispersion. For 48 hour acute studies, 14 days old *D. magna* were exposed at a density of 1 individual per 10 mL in each beaker for different concentrations. Three replicates were performed for each treatment and control groups were also assessed in the absence of LaCoO_3_. *D. magna* were not fed during the exposure period.

#### 2.3.2 Bioaccumulation of LaCoO_3_ in *D. magna*

*D. magna* were exposed to LaCoO_3_ (0, 5 and 50 ppm) and Co ions (0, 60 and 60 ppb) for 0, 3, 12, 24 and 48 h. The assessed Co ion concentrations of 60 and 600 ppb were selected based on the dissolution kinetics of LaCoO_3_ NPs in SM7 medium (data not shown). La was poorly dissolved.^25^ After treatment, 10 daphnids were collected at each time point and washed in EDTA-2Na and ultrapure water for a few minutes, to remove the spiked medium and the weakly adsorbed metals from their carapace. Daphnids were then dried at 80 °C to a constant weight and then digested in 68% nitric acid for 6 h. ICP-MS was used to determine the level of metal accumulation in *D. magna*, with accumulation calculated according to the dry weight of *D. magna* (μg g^−1^ d.w.).

#### 2.3.3 Determination of ROS in *D. magna*

The amount of ROS produced in *D. magna* was determined by 2,7-dichlorofluorescein (H_2_DCFDA), a type of ROS-sensitive fluorescent dye.^26^ Firstly, a 10 μmol L^−1^ DCFH-DA (Analytical grade; Amor, Inc., USA) solution was prepared. After exposed to 5 ppm and 50 ppm LaCoO_3_ for certain hours, five live daphnids were collected at each time point and washed in ultrapure water, then transferred to 3.5 mL DCFH-DA solutions. After 4 hours of incubation in the dark, daphnids were washed three times in ultrapure water. The tissues of daphnids were homogenized by ultrasonication in 0.5 mL of sucrose buffer (0.25 M sucrose, 0.1 M Tris–HCl, pH 8.6) and then centrifuged at 16 000 × *g* for 20 minutes. The supernatant was collected to determine the protein content and perform fluorescence measurements. Tissue protein content in *D. magna* were assayed using commercially available kits (Nanjing Jiancheng Bioengineering Institute, China) according to the manufacturer's protocol. Fluorescence (excitation at 485 nm and emission at 530 nm) was quantified using a spectrofluorometer (Hitachi, F7000, Japan). Fluorescence was expressed per tissue protein content, with data were expressed as a percentage as compared with the relevant negative control.

#### 2.3.4 Determination of SOD and Na^+^/K^+^-ATPase *D. magna*

Twenty surviving individuals were weighed after all water was dried from the surface of daphnids and tissues were homogenized according to the method defined in Section 2.3.3. The supernatant after centrifugation was used to determine SOD and Na^+^/K^+^-ATPase activities, which were assayed using a commercially available kit (Nanjing Jiancheng Bioengineering Institute, China) according to the manufacturer's instructions. SOD was determined based on the SOD-induced inhibition of NADPH oxidation by molecular oxygen.^27^ One unit of activity (U) was defined as the SOD amount per mg tissue protein when the inhibition rate reached 50%, in 1 mL reaction solutions. The determination of Na^+^/K^+^-ATPase was based on the amount of inorganic phosphorus produced by decomposition of ATP, catalyzed by ATPase. One unit of activity (U) was defined as the amount of inorganic phosphate produced through ATP decomposition by ATPase per mg tissue protein per hour. All enzymatic activities were calculated per mg of protein and data were expressed as a percentage activity as compared with the relevant negative control.

### 2.4 μ-XRF analysis: element mapping in *D. magna*

After exposure to 5 or 50 ppm LaCoO_3_ for certain hours, *D. magna* were washed for 10 min in EDTA-2Na and ultrapure water and then fixed using hexamethyldisilazane as described by Tan *et al.*^15^ μXRF analyses of La and Co were performed using a BL15U beamline at the Shanghai Synchrotron Radiation Facility (SSRF; China) which runs a 3.5 GeV electron beam with a current range from 200 to 300 mA. Element maps were obtained using stepwise analysis mode and the excitation X-ray beam was set at 100 μm × 100 μm with a living time of 3 s. Fluorescence data was processed using Igor and PyMca package software.

## 3. Results

### 3.1 Characterization

**Fig. 1:**
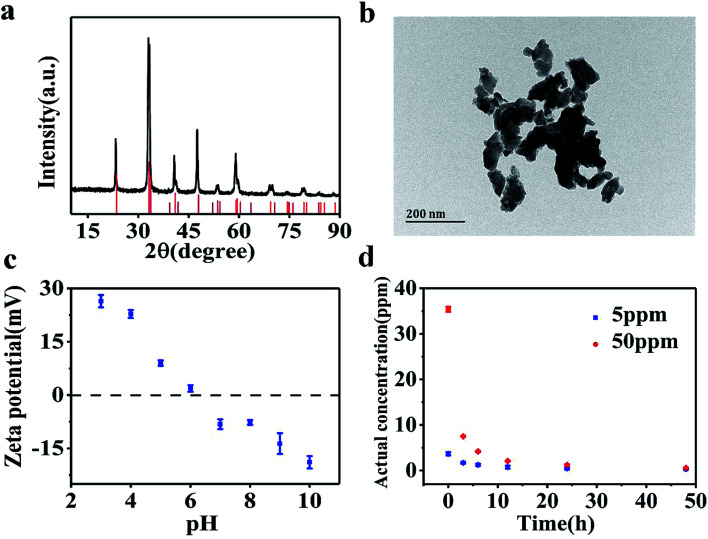
(a) XRD pattern of LaCoO_3_ NPs. (b) TEM image of LaCoO_3_ NPs. (c) Zeta potential of LaCoO_3_ NPs as a function of pH. (d) Actual concentration of LaCoO_3_ NPs in suspension.

Fig. 1a shows the XRD patterns of the synthesized LaCoO_3_ NPs. The major diffraction peaks observed are in agreement with PDF# 84-0847 of the JCPDS database cards, confirming the perovskite structure of the synthesized samples. The particle size of LaCoO_3_ NPs was determined as being 20–100 nm based on TEM imaging results (Fig. 1b). However, LaCoO_3_ NPs tended to aggregate in SM7 medium. The zeta potential values as a function of solution pH are shown in Fig. 1c for a 50 ppm LaCoO_3_ dispersion. The *ζ* potential values continuously decreased from +16.7 mV at pH 3 to −18.9 mV at pH 10. The point of zero charge (PZC) was observed at around pH 6 for MilliQ water. Fig. 1d illustrates the actual concentrations of LaCoO_3_ NPs detected in solutions with nominal concentrations of 5 and 50 ppm in suspension. The actual concentration results show that LaCoO_3_ NPs settled rapidly in SM7 medium, causing the concentration to decrease over time. By the end of the exposure period, the actual concentration for solutions with nominal values of 5 ppm and 50 ppm were 0.3 ± 0.07 and 0.5 ± 0.2 ppm, respectively.

### 3.2 Bioaccumulation of LaCoO_3_ NPs in *D. magna*

**Fig. 2:**
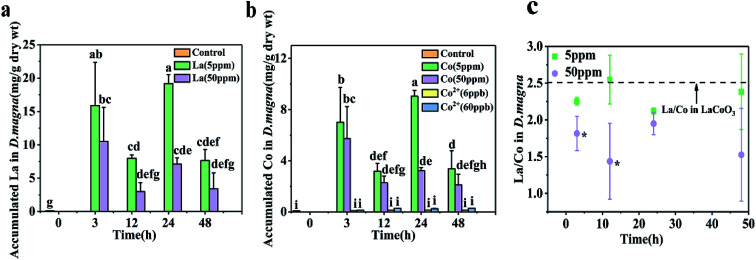
Concentrations of (a) La and (b) Co in *D. magna* during the 48 hour exposure to CoCl_2_ salts or LaCoO_3_, as measured by ICP-Ms. (c) The ratio of La and Co accumulated in *D. magna*. Data is expressed as a mean value ± standard deviation (*n* = 3). Different letters and asterisks indicate significant differences between the two treatments (*p* < 0.05).

*D. magna* were exposed to varying concentrations of CoCl_2_ and LaCoO_3_ for 48 h, with the amount of cobalt and lanthanum present within *D. magna* quantified by ICP-MS at the end of the defined exposure period (Fig. 2). In LaCoO_3_ NP exposed groups, in comparison with the control groups the amount of La and Co were statistically significantly increased (*p* < 0.05) in both 5 and 50 ppm exposure groups during the 48 h exposure period. A much higher cobalt concentration was observed in the LaCoO_3_ exposure group, compared to those exposed to the cobalt salt. In the 5 ppm exposed group at 3 h, the accumulated levels of La and Co were 15.9 ± 6.5 and 7.0 ± 2.7 mg g^−1^ dry weight, respectively. With extended exposure periods, the concentrations of La and Co reached a maximum at 24 h. After 48 h of exposure, large amounts of La and Co remained accumulated in daphnids, with 7.6 ± 1.6 mg La per g dry weight and 3.4 ± 1.4 mg Co per g dry weight. In 50 ppm exposed groups, at 3 h the accumulated La and Co concentrations were 10.5 ± 5.1 and 5.7 ± 2.5 mg g^−1^ dry weight, respectively. The concentrations of La and Co were significantly decreased at 12 h, compared with 3 h (*p* < 0.05). With longer exposure durations, the bioaccumulated concentrations of La and Co reached a steady state and after 48 h, the concentrations of La and Co were 3.4 ± 2.4 and 2.1 ± 0.8 mg g^−1^ dry weight, respectively. It is of note that the accumulated concentrations of La and Co were lower than the groups exposed to 5 ppm during 48 h exposure and significantly lower than 5 ppm exposure groups at 24 h.

Furthermore, the ratio of La/Co in daphnids was assessed during the exposure time. At the lower exposure concentration (5 ppm), the ratio of La/Co was close to that of pristine LaCoO_3_ and showed no change with time. However, at the higher exposure concentration (50 ppm), the ratio of La/Co was lower than that of the 5 ppm exposure group at 48 h, while being significantly lower than the 5 ppm exposure group at 3 and 12 h (*p* < 0.05).

### 3.3 Oxidative stress mediated by LaCoO_3_ NPs

**Fig. 3:**
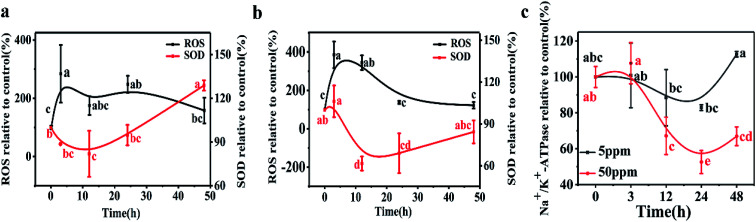
ROS and SOD in *D. magna* during 48 h exposure to LaCoO_3_ NPs: (a) 5 ppm (b) 50 ppm. (c) Na^+^/K^+^ ATPase activity in *D. magna* during 48 h exposure to LaCoO_3_ NPs. Data is expressed as a mean value ± standard deviation (*n* = 3). Different letters indicate significant differences between the two treatments (*p* < 0.05).

A time-dependent increase in ROS generation in *D. magna* was observed from 0 to 48 h in both exposure systems. In the 5 ppm exposure group, the *D. magna* ROS level rapidly increased and reached a maximum level (284% of the control) at 3 h. With further exposure, the ROS level gradually decreased and values at 48 h were similar to those of the control (*p* > 0.05). At the higher exposure concentration, ROS increased to 385% and 344% of the control groups at 3 and 12 h, respectively. Treatments showed no significant difference at 24 h and 48 h, than the initial values (*p* > 0.05) (Fig. 3).

In the 5 ppm treatment samples, SOD activities significantly decreased after 12 h exposure (*p* < 0.05) and then gradually increased. After 48 h exposure, the SOD activity was 120% of the control. However, in response to treatments with 50 ppm LaCoO_3_ NPs, although the SOD activity increased at 3 h, the increase was not significantly different from the initial value (*p* > 0.05). With further exposure, the SOD activity significantly decreased compared to the initial value at 12 and 24 h (*p* < 0.05). At 48 h, SOD levels were decreased to 82% as compared to the initial level.

The variation in Na^+^/K^+^-ATPase activity was found to be similar to that of SOD for both the low and high concentration exposure groups. Na^+^/K^+^-ATPase activity in daphnids exposed to 5 ppm LaCoO_3_ was not significantly affected compared to the initial value, during the whole 48 h exposure period. In response to exposure to 50 ppm LaCoO_3_, Na^+^/K^+^-ATPase activity was significantly decreased at the 12 and 24 h time point. With further exposure, the Na^+^/K^+^-ATPase activity gradually recovered, but it remained 30% lower than the initial value by 48 h (*p* < 0.05).

### 3.4 Localization of La and Co in *D. magna*

**Fig. 4:**
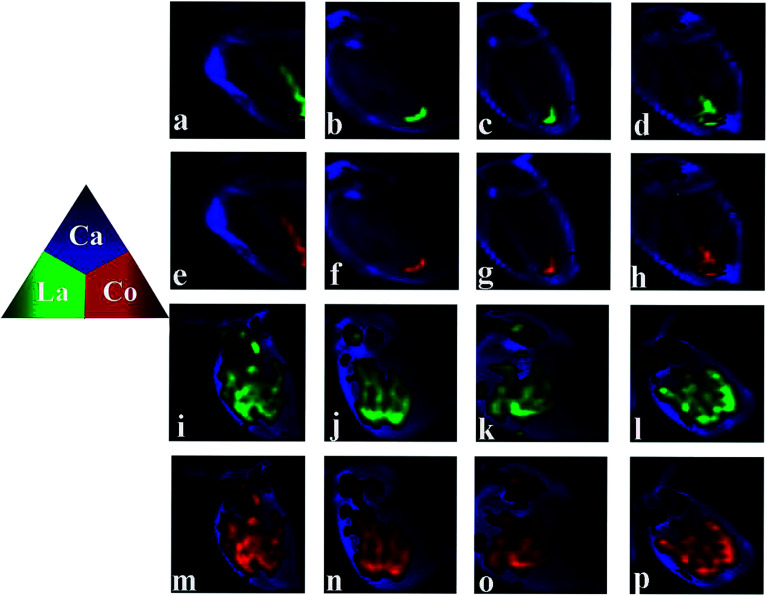
Distribution of Ca, La and Co in *D. magna* when exposed to LaCoO_3_ at 5 ppm (a–h) and 50 ppm (i–p) for 3 h (a, e, i, m), 12 h (b, f, j, n), 24 h (c, g, k, o) and 48 h (d, h, l, p). Images determined by synchrotron radiation-based micro X-ray fluorescence spectroscopy (μ-XRF).

The distribution of Ca is also presented, as Ca imaging provides a good proxy for the visual image of the organism. As can be seen from Fig. 4, daphnids rapidly take in LaCoO_3_ and after exposure to 5 ppm LaCoO_3_ for 3 h, a substantial amount of La and Co were found to have accumulated within daphnids, mostly in the midgut and hindgut regions, while relatively low concentrations were observed in the abdominal region. With longer exposure times (12–48 h), La and Co appeared to be predominantly localized to the hindgut and no change was observed with time. Furthermore, it is of note that the La and Co distributions were identical throughout the exposure time. In the 50 ppm treatment, the sites of La and Co accumulation were significantly different from the low concentration exposure, with La and Co distributed throughout both the gut and abdominal regions of the daphnids, but no significant La or Co signals were detected in the ovaries of daphnids at 50 ppm. In addition, the La and Co accumulation sites did not change with the exposure of time.

## 4. Discussion

Our result showed that in groups exposed to 5 ppm, La and Co located at the gut area, whereas both thoracic limbs and gut were the main target site for groups exposed to 50 ppm. In addition, it is interested to note that La and Co accumulated in daphnids exposed to 50 ppm was lower than that of 5 ppm. As for biomarker responses, significant oxidative stress were induced at both concentrations. In addition, high LaCoO_3_ also lead to the decrease of activity of the Na^+^/K^+^-ATPase, which is a conserved membrane enzyme located at the thoracic limbs and actively involved in ion regulation. This implies that different levels of LaCoO_3_ may have different modes of action. It is well known that both NPs and metal ions could induced oxidative stress in daphnids. In contrast, although some NPs were observed inhibited Na^+^/K^+^-ATPase activity, the inhibition effects of NPs are much less severe than metal ions. Thus we concluded that different action modes of LaCoO_3_ at lower and higher concentrations may related to the species of metal in daphnids. Different metal species enter the body of daphnids in different ways. As for LaCoO_3_, both aggregated and free ions existed in exposure medium, so it may involve different ways of entering daphnids, including dietary and waterborne.

### 4.1 Different mode of LaCoO_3_ accumulation at low and high concentrations

In the LaCoO_3_ exposure medium, a fraction of the particles readily dissolves, while some NPs form NP aggregates with an average size larger than 1 μm. This aggregation behavior may be due to the high ionic strength of the exposure medium and the pH of exposure medium being close to the PZC of LaCoO_3_ NPs. Therefore, when daphnids are added to the exposure medium, they are exposed to both LaCoO_3_ aggregates and free ions.

In the present study, the measured total Co concentration in daphnids exposed to LaCoO_3_, was much higher than for those exposed to the Co salt (Fig. 2). This indicates that particles play a significant role in the accumulation process, which is in accordance with the findings of Xiao *et al.*, and Adam *et al.*, showing that daphnids ingest more metals in particle form than in ion form.^28,29^

As a model organism in the aquatic food chain, *D. magna* can accumulate pollutant through three potential pathways. First, the particles of nanosize can enter the cells *via* endocytosis.^15^ Second, *D. magna* may feed on particles 0.4–70 μm in size, including the LaCoO_3_ NP and aggregates used in the current study, and accumulated in the gut region,^30^ namely dietary assimilation. Finally, the dissolved metal ions with a subnano size may be absorbed from the aqueous phase by organisms, the main route of entry is the epipodites of the thoracic limbs.

In the 5 ppm exposure group, the mass ratio of La and Co in daphnids were nearly 2.5 : 1, which is the ratio in the pristine LaCoO_3_ NPs. In addition, La and Co colocalized accumulated in the midgut and hindgut at the beginning of exposure. This indicating that both these metals existed in LaCoO_3_ particle form in daphnids and ingested by daphnids through dietary way. With extended exposure periods, LaCoO_3_ were found to be localized only in the hindgut. This phenomenon may partly be due to the physiology of daphnids, with food rapidly passing through the gut of daphnids and transferring from the midgut and accumulating in the hindgut, before being finally evacuated.^31^ In addition, due to the high ionic strength and pH of the exposure medium, large LaCoO_3_ aggregates were formed. The concentration of LaCoO_3_ in exposed medium decreased as the exposure time increased, implying that most LaCoO_3_ precipitated from solution over time, which may lead to the net release of accumulated LaCoO_3_ by daphnids. A decrease in accumulation by daphnids has also reported in the uptake of Au NPs,^32^ Fe_2_O_3_ NPs^33^ and carbon nanotubes.^34^ However, LaCoO_3_ exhibited a long gut residence time and at the end of the exposure period, a high volume of LaCoO_3_ remained in the gut of daphnids (0.76% *D. magna* d.w. of accumulated La and 0.34% *D. magna* d.w. of accumulated Co), indicating that LaCoO_3_ elimination by daphnids is difficult. This body burden is similar to the accumulation reported in daphnids exposed to CNMs (0.7%)^35^ and graphene (0.7%).^36^ Previously studies have shown that perovskite agglomerates accumulate in the intestine of daphnids and may affect food intake and cause chronic toxicity.^25^ Also, persistent NP accumulation in organisms may cause negative impacts, such as organ injury or biomarker dysfunction.^37^ Considering that *D. magna* is a lower trophic level organism in the freshwater food chain, this observed persistence suggests that the accumulation of LaCoO_3_ may not only have long-term adverse effects for the exposed daphnids, but may also present an additional risk for the whole ecosystem.

However, at high exposure concentrations, the accumulation and distribution of LaCoO_3_ appear to differ from that of lower concentration exposures. The concentrations of total La and Co accumulated in daphnids were not increased with the exposure concentration, less total La and Co were found in 50 ppm than that in 5 ppm. We supposed this was due to large amount of free ions accumulated in daphnids except for LaCoO_3_ particle at 50 ppm exposed groups because of the following reasons. Although the proportion of dissolved NPs were the same at both LaCoO_3_ concentration exposure medium, lower mass ratio of La and Co values were observed in daphnids exposed to 50 ppm LaCoO_3_ than those exposed to 5 ppm, which indicated that more free Co ions were accumulated in daphnids exposed to high LaCoO_3_ concentrations in addition to LaCoO_3_. In addition, the distribution map of La and Co in 50 ppm exposed groups, which is distinct different from that of 5 ppm. La and Co was distributed not only in gut but also in the thoracic limbs. Knowing the fact that LaCoO_3_ formed aggregate larger than 1 μm, the possibility that La and Co in thoracic limbs was assimilation of LaCoO_3_*via* endocytosis was excluded. We think that La and Co distributed in thoracic limbs was La ions and Co ions through waterborne uptake and the signal in the gut area was LaCoO_3_ particle through dietary assimilation. Because of the accumulated free ions, the uptake of LaCoO_3_ particles was inhibited. This was supported by the idea that an increased body burden of Co ions will cause general impairment of biological activities, including feeding.^38^ Feeding inhibition has been observed in freshwater snails exposed to Co ions and the inhibition effect was due to impairment of the uptake and homeostasis of Ca.^38^ Although Co is an essential metal for all living organisms, toxicity occurs during exposure to elevated concentrations. The primary toxic effect induced by Co has been proposed to be impairment of Ca uptake, as both Co and Ca share similar uptake pathways.^38,39^ Thoracic limbs play a crucial role in food particle collection. Due to the depletion of Ca^2+^, muscle contraction and thoracic limb activity is inhibited, leading inevitably to a decrease in food uptake.^40^ As a result, the uptake of LaCoO_3_ at 50 ppm was lower than with exposure to 5 ppm LaCoO_3_.

### 4.2 Different toxicity mechanism at high and low concentrations of LaCoO_3_

**Fig. 5:**
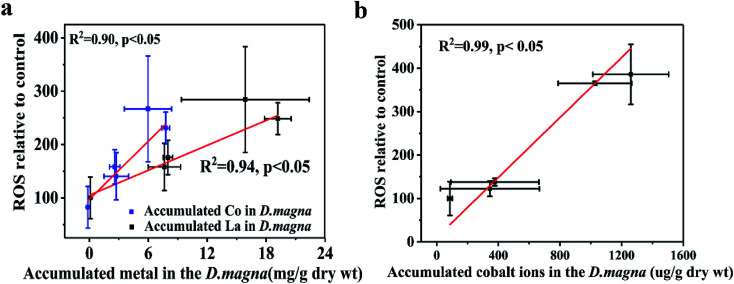
Relationships between ROS and accumulated metals in *D. magna*. (a) 5 ppm LaCoO_3_ (b) 50 ppm LaCoO_3_. Mean ± standard deviation (*n* = 3).

We analyzed the trends in biomarker responses to elucidate the antioxidant processes involved in LaCoO_3_ exposure. LaCoO_3_ induces significant changes in ROS levels and *D. magna* enzyme biomarkers. These results indicate that LaCoO_3_ accumulated in the intestine or abdominal region, can produce ROS through various biochemical processes. However, although the concentration of total La and Co in daphnids was higher in the 5 ppm exposed group than the 50 ppm, more ROS and a greater degree of oxidative stress were induced at higher concentrations. Therefore, the relationship between ROS production in daphnids and metal accumulation was investigated (Fig. 5). Results show that there was a significant positive correlation between the growth of ROS and the accumulation of La and Co at 5 ppm (*p* < 0.05). As the levels of La/Co in daphnids were similar to the levels observed in pristine LaCoO_3_ NPs, it is likely that ROS increased in accordance with greater accumulation of LaCoO_3_ particles in daphnids. A similar correlation was observed in daphnids exposed to titanium dioxide, with ROS production increasing with accumulation of TiO_2_.^13^ At high concentration exposure levels, the relationship between ROS and total accumulated La or Co was not observed, indicating that the mechanism of ROS induction is different at higher concentrations than at lower concentrations. As a higher proportion of Co were observed at 50 ppm, the level of Co ion accumulation in daphnids was calculated by subtraction, showing that Co ions are positively correlated with ROS produced at high LaCoO_3_ concentrations (*p* < 0.05). Co ions are biologically essential elements, which have a very important role in many physiological processes.^39^ However, ROS can be catalyzed by high concentrations of Co *in vivo*, leading to oxidative stress. Rapid production of ROS has been observed in cells exposed to Co-containing NPs such as Co_3_O_4_.^41^ In addition, the contribution of particles to the production of ROS should not be excluded, as a large number of LaCoO_3_ particles accumulated in the organisms filter apparatus. Filter apparatus are thought to be a highly susceptible organ to NP exposure, with the generation of ROS more evident in *D. magna* filter apparatus, than the intestine.^14^ Therefore, it may be inferred that the higher levels of ROS induced in the 50 ppm exposure group, was partly because ROS was induced to a greater degree in the filter apparatus.

Organisms can adapt to increased levels of ROS production by regulating antioxidant defenses, such as the activities of antioxidant enzymes. SODs act as a first line of defense against ROS, regulating overall ROS levels to maintain physiological homeostasis.^42^ If ROS levels continue to increase, the antioxidant defenses can be overwhelmed and excessive ROS result in protein denaturation, membrane damage and DNA damage.^41^

The variation tendency of biomarkers was assessed in the present study, to elucidate the common antioxidant responses to LaCoO_3_ exposure at low and high concentrations. It was found that the oxidative stress mechanism induced by LaCoO_3_ could be divided into 2 stages: oxidation inhibition and antioxidative recovery. The oxidation inhibition stage refers to the first 12 hours of exposure, when abnormally high levels of ROS were generated rapidly and the enzymatic mechanisms were inhibited to some extent and a significant decrease in SOD was observed. This finding is different from previously reported studies, which have indicated that SOD is induced in the first stage of exposure.^26,43^ The observed decrease in SOD may be associated with direct damage to SOD after LaCoO_3_ exposure. Previous studies have shown that Co ions can reduce the activity of SOD by accommodated in copper or manganese-binding active site in CuZn-SOD and Mn-SOD, respectively.^44,45^ The second stage of antioxidant recovery, occurs between 12 and 48 h of exposure, where due to the continuous work of antioxidative defense systems, excessive ROS levels are decreased and eventually eliminated completely. The various different physiological functions of the body, for example, the activity of SOD, recovered gradually although at the end of the exposure period they remained at a lower level than the control groups.

Na^+^, K^+^-ATPase has been found in a variety of tissues from different animals including aquatic organisms, which is located on the cell membrane and plays an important role in the process of cellular ion transport and energy metabolism at the expense of ATP.^46^ There are documented inhibitory effects on Na^+^/K^+^-ATPase in aquatic organism of heavy metals such as Ag^+^, Cu^2+^, Zn^2+^. Present study showed that Na^+^/K^+^-ATPase was inhibited significantly after exposed to LaCoO_3_ at 50 ppm. In contrast, while no obvious effect observed at 5 ppm LaCoO_3_. This may be due to the fact that lanthanum ions and cobalt ions accumulated in daphnids at higher concentrations interfere with ion transport and membrane permeability through Ca^2+^ channel,^47,48^ while particle accumulation showed weak impact. Similar effect was observed by Griffitt *et al.*^49^ that the inhibition of Na^+^/K^+^-ATPase by nano-Cu was not as great as dissolved Cu and it is reported that the inhibition effects of Na^+^/K^+^-ATPase of dietary metal exposure are much less severe than *via* the aqueous route.^50^ It is well-known that ions uptake of adult daphnids occurs primarily in the ion-transporting regions of the epipodites, gill like lamellar surfaces located at the base of the thoracic limbs.^51,52^ The result of μ-XRF of present study manifested that large number of lanthanum ions and cobalt ions located in the thoracic limbs at 50 ppm exposed groups, suggesting the possible ions transport interference at thoracic limbs. On the other hand, cobalt, in the ionized species Co^2+^, is able to suppresses synthesis of ATP and the reduction in ATP production, in turn, alters Na^+^/K^+^-ATPase activity.^53,54^ Additionally, Na^+^/K^+^-ATPase can be very sensitive to oxidative damage and decreased Na^+^/K^+^-ATPase activity with altered antioxidant enzyme activities *in vivo* were observed.^54,55^ In our study, Na^+^/K^+^-ATPase activity significantly decreased in the same manner as SOD, although the impairment of Na^+^/K^+^-ATPase activity was delayed as compared with SOD. In this sense, due to the reduced protection by SOD, the excessive ROS production can attack polyunsaturated fatty acid in the biomembrane, leading to its dysfunction and thereby may impairing the structure of Na^+^/K^+^-ATPase, which explain its inhibition.

## 5. Conclusion

We have provided the first detailed investigation of mechanism of LaCoO_3_ as a representative PNM. Result showed that the mode of action of LaCoO_3_ was dependent Co form, which resulted in the differences in the respect of uptake, accumulation, distribution and toxicity mechanism. At low exposure concentrations, LaCoO_3_ particle can be rapidly ingested through dietary exposures and was mainly located in the intestine and incompletely depurated by daphnids. However, at high exposure concentrations, more attention should be paid to the dissolved part of LaCoO_3_ in the exposure medium. La and Co accumulated at thoracic limbs and gut from both dissolved and dietary exposures and inhibit the activity of Na^+^/K^+^-ATPase. Moreover, both levels of LaCoO_3_ triggered oxidative stress, which correlated to the form of Co accumulated in daphnids, too. Although the concentrations of LaCoO_3_ used in the present investigation may be higher than the environmental level, the findings may contribute the knowledge on the toxicity of nano-perovskite on aquatic organisms for which the data are very limited.

## Conflicts of interest

There are no conflicts to declare.

## Supplementary Material
